# Survival of *Pseudomonas syringae* pv. *actinidiae* in detached kiwifruit leaves at different environmental conditions

**DOI:** 10.7717/peerj.15031

**Published:** 2023-03-10

**Authors:** Siyi Deng, Wei Chang, Yawei Que, Jun Liu, Hua Wang

**Affiliations:** 1 Institute of Plant Protection and Soil Fertilizer, Hubei Academy of Agricultural Sciences, Wuhan, Hubei, China; 2 Key Laboratory of Integrated Pest Management on Crops in Central China, Ministry of Agriculture, Wuhan, Hubei, China; 3 Hubei Key Laboratory of Crop Disease, Insect Pests and Weeds Control, Wuhan, Hubei, China

**Keywords:** Kiwifruit canker, *Pseudomonas syring* pv. *actinidiae*, Infection path, Survival time, Population growth, Temperature and humidity

## Abstract

*Pseudomonas syringae* pv. *actinidiae* (*Psa*) is the causal agent of kiwifruit canker, a serious threat to commercial kiwifruit production worldwide. Studies of the movement path and the survival time of *Psa* in the host are crucial for integrated management programs. Hence, we used *Psa* with GFPuv gene (*Psa-*GFPuv) strain to investigate the movement path of *Psa* in leaves and branches, and the survival time of *Psa* in leaves under different environmental conditions. We found that the pathogen *Psa* spread longitudinally in the branches and leaves rather than transverse path. Additionally, the survival time of bacteria in fallen leaves under different environmental conditions were simulated by the way of *Psa* infecting the detached kiwifruit leaves. *Psa* survives the longest, up to 43 days in detached kiwifruit leaves with high humidity (above 80%) at 5 °C, and up to 32 days with low humidity (20%). At 15 °C, the *Psa* can survive in detached kiwifruit leaves for 20–30 days with increasing humidity. At 25 °C, it can only survive for 3 days with low humidity (20%) and 15 days with high humidity (above 80%). Furthermore, the population growth experiments showed that bacterial growth of *Psa* was more favorable in detached kiwifruit leaves with above 80% humidity at 5 °C. These results suggest that the survival condition of *Psa* in detached kiwifruit leaves is significantly affected by environmental conditions, and provide the basis for the control timing and technology of kiwifruit canker.

## Introduction

Kiwifruit belongs to the *Actinidia*, which is derived from a deciduous, woody fruiting vine (Huang & Ferguson, 2007). Red heart kiwifruit such as ‘Hongyang’ has developed rapidly and has strong market competitiveness (Wang et al., 2020; Pereira et al., 2021). However, red heart kiwifruit is highly susceptible to kiwifruit canker, which has become an increasingly serious and destructive disease, seriously affecting the production and development of kiwifruit (Kim et al., 2016b).

Kiwifruit canker has the characteristics of wide range, rapid spread, strong pathogenicity and difficult control, which is easy to cause large-scale death of trees in a short time. Kiwifruit canker was first occurred in 1983 on a green fleshed kiwifruit cultivar (*Actinidia deliciosa*), Hayward, in Japan (Serizawa et al., 1989). Then, this pathogen has been reported in the United States (Scortichini et al., 2012), South Korea (Koh et al., 2010), New Zealand (Everett et al., 2011), Italy (Balestra et al., 2009), France (Vanneste et al., 2011b), and China (Fang, Zhu & Wang, 1990). In China, kiwifruit canker first occurred in Dongshanfeng farm, Hunan Province, and 133.3 hm^2^ kiwifruit orchards were destroyed in 3 years (Fang, Zhu & Wang, 1990). At present, the kiwifruit canker has occurred in the kiwifruit planting areas of 16 provinces in China (Zhong et al., 2020). The average incidence rate is about 20%, and the incidence rate of severe orchards has exceeded 70% (Li et al., 2013), which is increasing year by year, seriously hindering the yield and income of the kiwifruit industry.

Kiwifruit canker caused by *Pseudomonas syringae* pv. *actinidiae* (*Psa*) can be divided into two periods (Serizawa et al., 1989; Everett, 2011). In the first phase during late winter, the rapid growth of *Psa* and the hydration and swelling of bacterial exopolysaccharides blocked the xylem vessels, and the water pressure caused by the end of dormancy may lead to the cracking and exudation of the trunk, leaders and canes (Kim et al., 2016b). In the second phase during late winter or early spring, environmental conditions are very favourable for disease development and primary infection can be facilitated by physical damage caused by frost, high winds and rain (Serizawa et al., 1989). In China, during the late winter in February or early spring in March, natural openings or trimmed wounds of canes and trunks often shed milky bacterial secretions. Milky bacterial secretions are an important source of pathogens transmitted by heavy rainfall and strong winds (Serizawa et al., 1989; Ferrante et al., 2012; Son et al., 2016). In spring, *Psa* infects flowers, leaves and fruits through stomata, hydathodes, lenticels and wounds (Serizawa & Ichikawa, 1993; Spinelli et al., 2011; Marcelletti et al., 2011; Ferrante et al., 2012). Previous reports showed that infected pollens, asymptomatic flowers and leaves, leaves litter, and pruning debris may be important inoculation sources of *Psa* (Stefani & Giovanardi, 2011; Vanneste et al., 2011a; Vanneste et al., 2011b; Tyson et al., 2012; Kim et al., 2016a). Moreover, the rapid spread of *Psa* is mainly due to the use of contaminated scissors to infect plant wounds in winter (Koh et al., 2010; Everett, 2011; Kim et al., 2016b).

Serizawa & Ichikawa (1993) pointed out that the climate of rainy, high humidity and low temperature (12–18 °C) in early spring are conducive to the rapid proliferation of *Psa*, and the harm of pathogens is weakened when the temperature rises to 25 °C. In addition, previously reported that low temperature was conducive to colonization and movement of *Psa*-GFPuv in kiwifruit tissues (Gao et al., 2016). There are also many studies showing that the disease is more likely to be prevalent at low temperature and high humidity (Li et al., 2001; Han et al., 2013). However, the infection pathway of *Psa* in kiwifruit plants and the survival time under different environmental conditions are still unclear. Therefore, we explored the infection path of kiwifruit canker in the plant, and the survival time of *Psa* in detached kiwifruit leaves under different environmental conditions. The survival time of bacteria in fallen leaves under different environmental conditions were simulated by the way of *Psa* infecting the detached kiwifruit leaves, to clarify the way of *Psa* infecting kiwifruit roots by soil-borne. Our findings would provide the foundation for the control and field management research of kiwifruit canker in the future.

## Materials and Methods

### Bacterial strains and plants

The bacterial strain *Pseudomonas syringae* pv. *actinidiae* was obtained from the Wuhan Botanical Garden, Chinese Academy of Sciences, and belongs to the biovar three clade of the *Psa* phylogeny. The method of GFPuv protein transformed into the *Psa* strain as described previously (Wang et al., 2017). *Psa-*GFPuv strain was cultured in King’s medium B (KB) liquid medium overnight at 28 °C with shaking at 220 rpm, and adjusted to a concentration of about 1 × 10^9^ CFU/ml with sterile double-distilled water. Bacterial growth was monitored by measuring the absorbance of cell suspensions at 600 nm. The variety of kiwifruit used is “Hongyang” red heart kiwifruit.

### The symptoms of kiwifruit canker in the field

The kiwifruit in Wuhan, Shiyan, Danjiangkou, Huanggang, Xiangyang, and other main planting areas in Hubei Province, China were surveyed to determine if kiwi canker symptoms appeared in orchards from 2021 to 2022. We observed a total of eight orchards infected or suspected of being infected by the bacterial canker to investigate the disease.

### Kiwifruit branch inoculation assay

Firstly, the healthy branches were cut with sterile scissors, and disinfect the round wound surface of the branches. Then, for inoculation assay, the *Psa*-GFPuv strain was collected by centrifugation and adjusted to a concentration of about 1 × 10^9^ CFU/ml with sterile double-distilled water. *Psa-*GFPuv strain was inoculated into both ends of the branch using a sterilized toothpick. The inoculated kiwifruit branches were incubated in an artificial climate box (LongYue, ShangHai) with a relative humidity (RH) of 100% at 25 °C. The infection process of *Psa-*GFPuv in kiwifruit branches was observed at 10 days post-inoculation (dpi) under UV light (395 nm). The experiments were performed three times, with three biological replicates per treatment.

### Kiwifruit leaves inoculation assay

The *Psa-*GFPuv bacterial suspension is pricked into the veins of kiwifruit leaves using sterile toothpicks (bacterial suspension concentration is 1 × 10^9^ CFU/ml). Seven inoculation points were selected for each leaf. Three kiwifruit leaves of similar size and growth are served as a group. The inoculated kiwifruit leaves were incubated in an artificial climate box (Longyue, Shanghai, China) with a relative humidity (RH) of 100% at 25 °C. The infection process of *Psa-*GFPuv in kiwifruit leaves was observed at 10 dpi under UV light (395 nm). The experiments were performed three times, with three biological replicates per treatment.

### Survival assay of *Psa-*GFPuv on detached kiwifruit leaf under different environmental conditions

*Psa-*GFPuv strains were collected by centrifugation and adjusted to a concentration of about 1 × 10^9^ CFU/ml with sterile double-distilled water. All kiwifruit leaves used for inoculation were taken from healthy kiwifruit trees at the kiwifruit orchard in Wuhan, Hubei province. Then, *Psa-*GFPuv strains was pricked into the veins of 30 kiwifruit leaves using sterile toothpicks. Seven inoculation points were selected for each leaf. Three kiwifruit leaves of similar size and growth are served as a group. The survival time of kiwifruit leaves inoculated with *Psa* at 5 °C, 15 °C and 25 °C was determined by observing the disappearance time of green fluorescence of the strain. In addition, four humidity (20%, 50%, 80% and 100%) were set under each temperature condition. Adjust the environmental conditions through the artificial climate box (Longyue, Shanghai, China). The experiments were performed three times, with three biological replicates per treatment.

### Bacterial growth assay of *Psa-*GFPuv in detached kiwifruit leaves under different environmental conditions

The bacterial growth of *Psa-*GFPuv strain at different environmental conditions was determined by inoculating kiwifruit leaves. The method of *Psa* injection the kiwifruit leaves as above. The quantitative analysis method of bacterial growth as show as Fig. S1. The method of bacterial populations growth in plants as previously described in Liu et al. (2022). Briefly, bacterial cells were recovered from plants by taking three samples from three leaves at the site at different days postinoculation (dpi). Since there were many inoculation time points for collecting leaf samples, the samples collected at each inoculation time were all from new leaves, and the same three kiwifruit leaves were not sampled repeatedly. The leaf disks were then crushed and put into a centrifuge tube with 100 μl of PBS. The homogenate was serial 10-time diluted and plated on KB plates. After incubating at for different days, and the number of CFUs per disk (cm^2^) was calculated. The experiment was repeated three times.

### Statistical analyses

SPSS statistics version 26 (IBM, Armonk, NY, USA) was used to perform a one-way ANOVA and least significant difference (LSD) test to determine the significant difference in the survival time and bacterial colonization of *Psa* in *detached* kiwifruit leaves at different environmental conditions. Differences with *P* < 0.05 were considered statistically significant.

## Results

### Symptoms of kiwifruit canker

**Figure 1:**
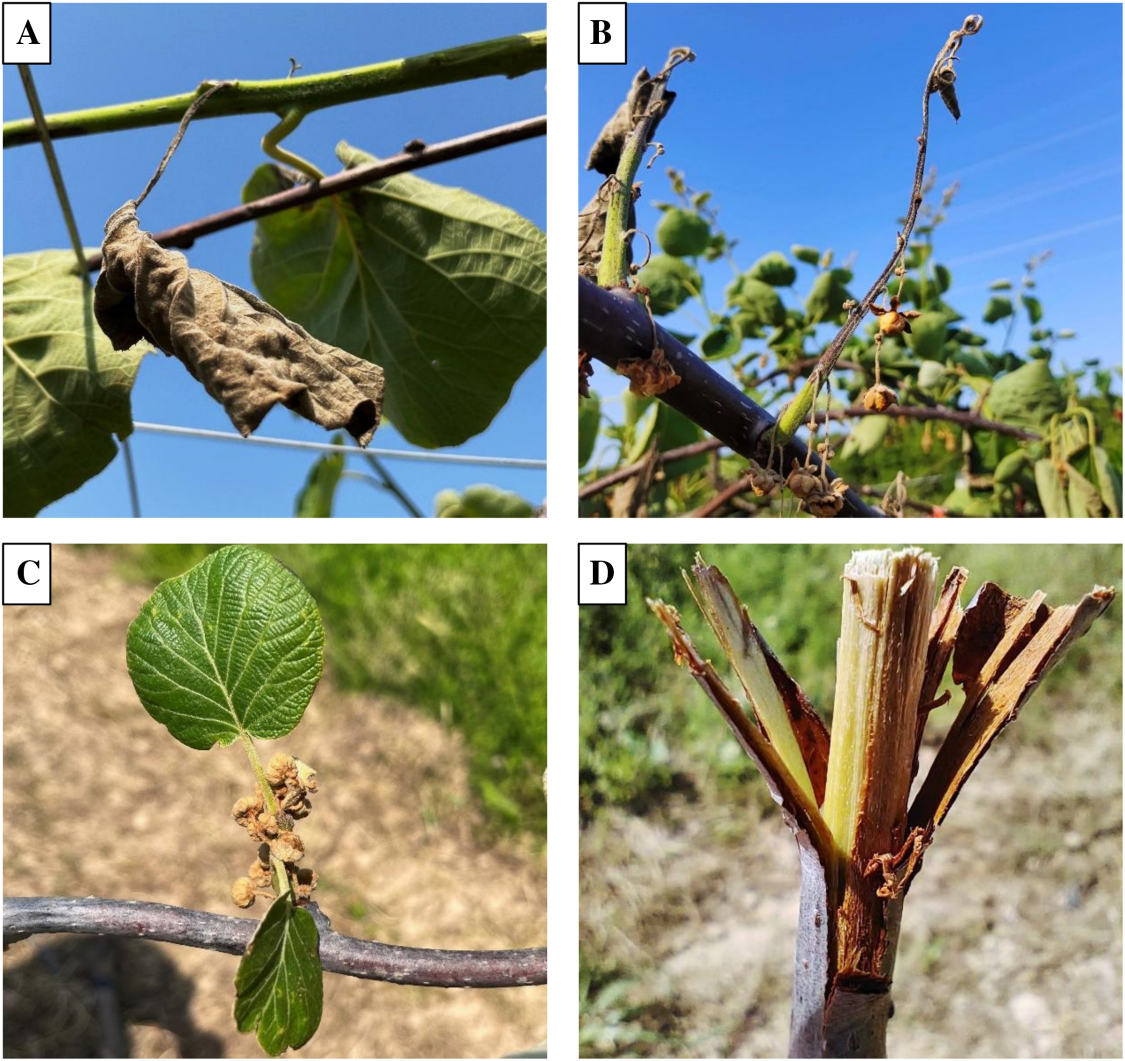
Typical symptoms of kiwifruit canker in kiwifruit orchard. (A) Kiwifruit canker symptoms on leaves. (B) Kiwifruit canker symptoms on shoots. (C) Kiwifruit canker symptoms on flowers. (D) Kiwifruit canker symptoms on branches.

The kiwifruit canker was observed at kiwifruit orchards of Wuhan, Shiyan, Danjiangkou, Huanggang, Xiangyang, and other main planting areas in Hubei Province in 2021 and 2022. After observing a large number of kiwifruit canker symptoms, we generally found that part of the plant have symptoms, while adjacent tissues could grow normally. As is shown in Figs.1A and 1B, the leaves and the shoots were completely necrotic, while the branches connected to them were healthy, and the other leaves and shoots on the branches were not affected either. Similarly, flowers were completely affected while leaves growing from the same branch were healthy (Fig. 1C). In addition, symptoms on branches can be seen as evidence that the infection was longitudinal rather than transverse (Fig. 1D).

### *Psa-*GFPuv spreads linearly in the kiwifruit branches

**Figure 2:**
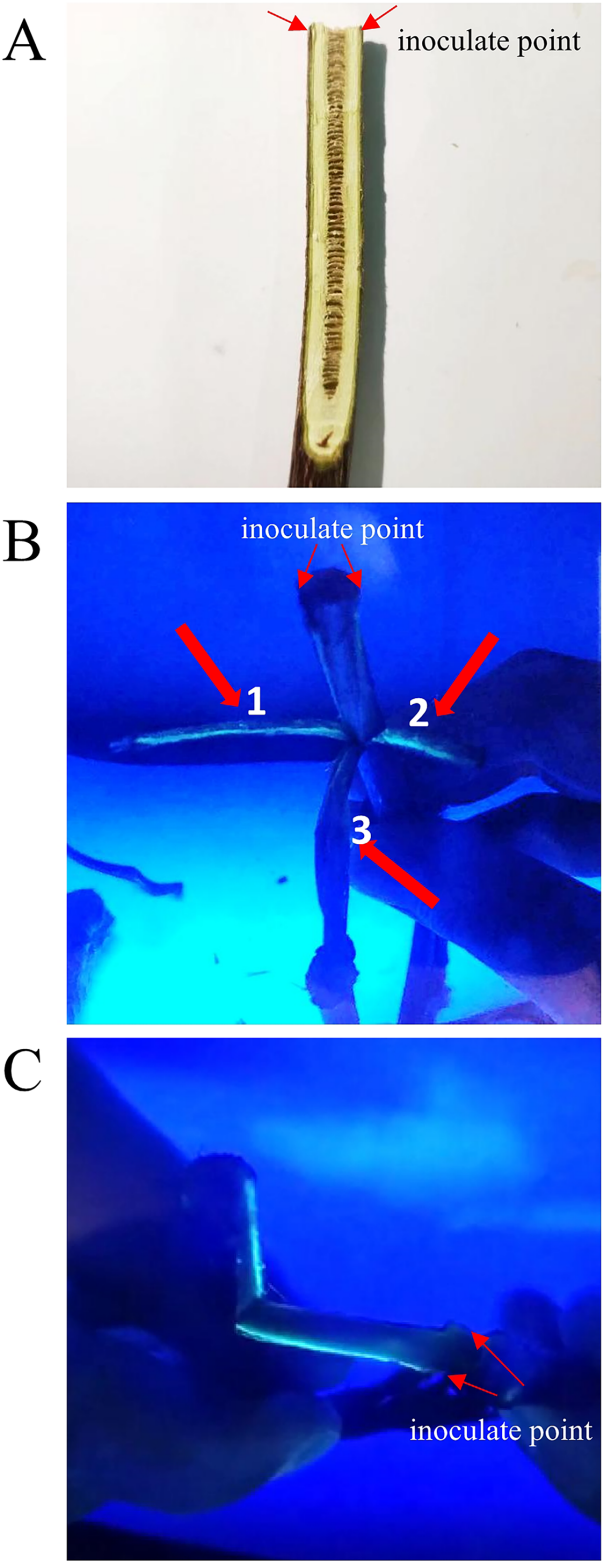
Infection path of *Psa-*GFPuv inoculated kiwifruit branches. (A) The visible light photograph of *Psa*-GFPuv inoculated kiwifruit branches. (B) Aerial view of the *Psa-*GFPuv infection pathway. 1 and 2: *Psa-*GFPuv spreads linearly along kiwifruit branches; 3: Between *Psa-*GFPuv inoculate points. (C) Lateral view of the *Psa-*GFPuv infection pathway.

Linear transmission of *Psa* along the phloem of kiwifruit branches was observed in the field. In order to clarify the transmission characteristics of *Psa* on kiwifruit branches, we conducted inoculation experiments on kiwifruit branches as show as Fig. 2A. The results of inoculation of kiwifruit branches with *Psa-*GFPuv showed that green fluorescence appeared in the annular wound, but did not spread laterally along the annular wound (Fig. 2B). The inoculation site was cut with surgical scissors, and then the branch epidermis was pulled along the notch. A green fluorescent line can be seen from the inoculation point along the sieve tube on the torn epidermis (Fig. 2C). These results suggested that the *Psa-*GFPuv spreads linearly along plant tissue when infecting plant branches and the spread of pathogens was hindered by plant tissue.

### *Psa-*GFPuv spreads along the leaf vein in the kiwifruit leaves

**Figure 3:**
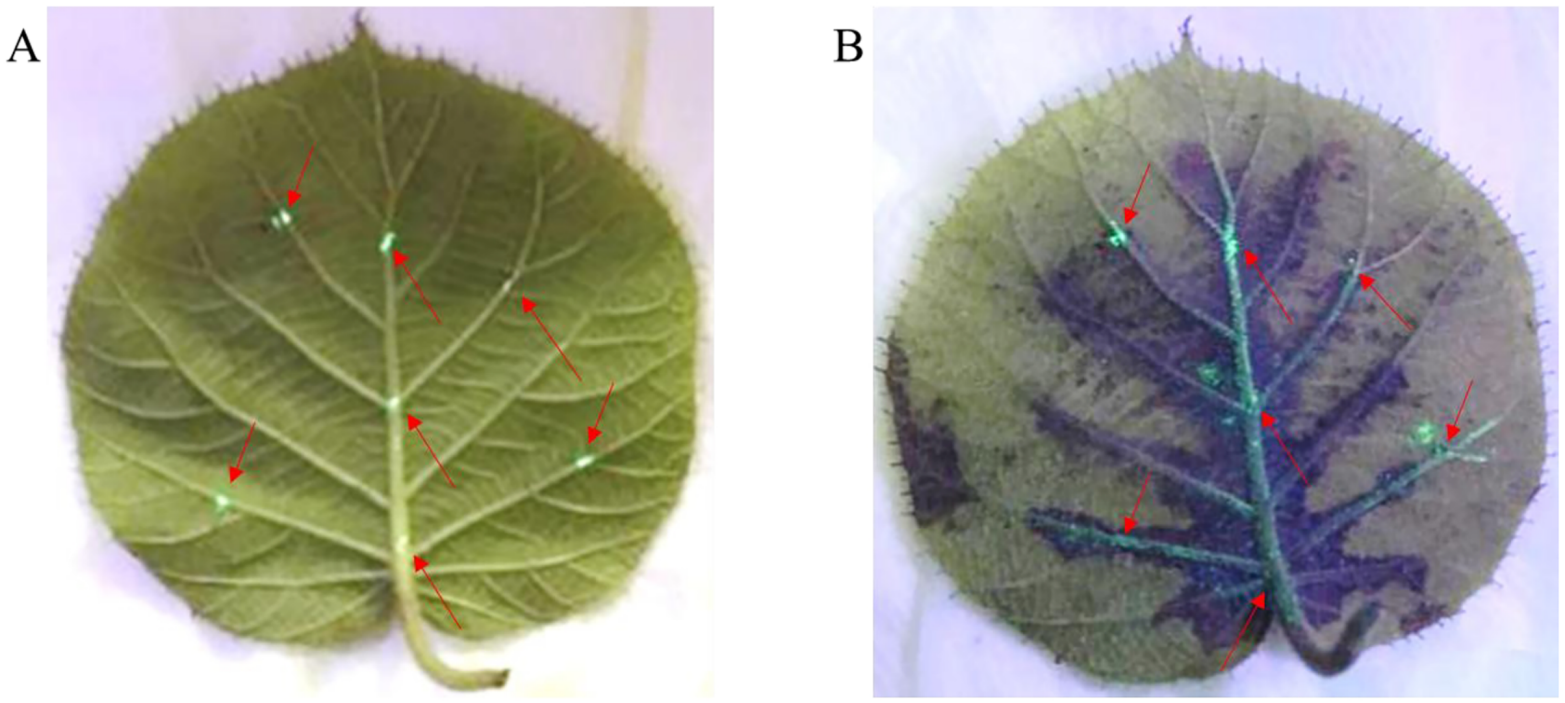
Infection path of *Psa-*GFPuv inoculated kiwifruit leaves. (A) The inoculation of *Psa-*GFPuv onto leaves at 0 dpi. (B) The infection of *Psa-*GFPuv into leaves at 4 dpi. The arrows represent the inoculated points on the leaf.

The mesophyll tissue of *Psa* inoculated leaves hardly showed the phenomenon of the spread of disease spots, indicating that *Psa* infection and diffusion in leaves may mainly occur in the vein. Therefore, to observe the survival status and movement of *Psa* in leaves, we conducted the vein inoculation experiment. As shown in Figs. 3A and 3B, the *Psa-*GFPuv was grow of colonization along the leaf veins and could not diffuse into mesophyll tissue. The kiwifruit leaf veins have obvious green fluorescence at 4 dpi, while the mesophyll tissues have no obvious green fluorescence Under UV light (395 nm) (Fig. 3B). This result showed that the spread of *Psa-*GFPuv on the kiwifruit leaves was hindered by plant tissue.

### *Psa-*GFPuv can survival around 40 days in detached kiwifruit leaves with suitable environment

**Figure 4:**
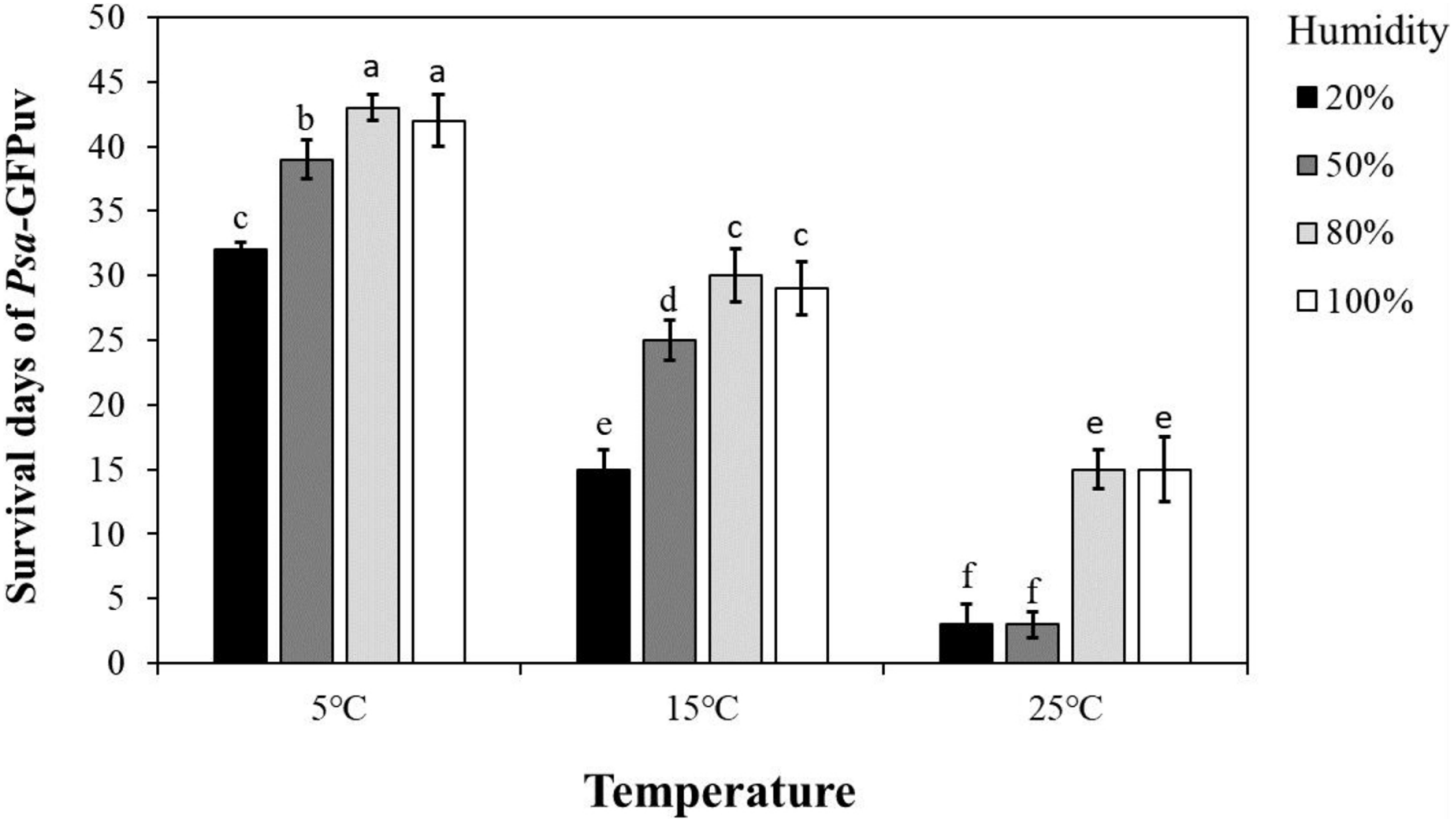
Survival days after *Psa*-GFPuv inoculated leaves at different temperatures and humidity. Different lowercase letters indicate a significant difference between treatments. Error bars indicate standard deviations. Statistically significant differences were determined by the one-way ANOVA of variance and *P* < 0.05.

To determine the survival time of *Psa* in kiwifruit leaves under different environmental conditions, we conducted kiwifruit leaves inoculation assay and found that *Psa-*GFPuv can survive for 32–43 days in kiwifruit leaves at 5 °C, 15–29 days at 15 °C, and 3–15 days at 25 °C (Fig. 4). Among them, at the same temperature, the survival time of pathogens in detached kiwifruit leaves with humidity above 80% was significantly longer (*P* < 0.05) than that of kiwifruit leaves with humidity below 50%. (Fig. 4). In addition, at the same humidity, the survival time of pathogens in detached kiwifruit leaves at 5 °C was significantly longer (*P* < 0.05) than that of kiwifruit leaves at 15 °C or 25 °C. These results indicate that environmental conditions play an important role in the survival of *Psa* in detached kiwifruit leaves.

### Bacterial growth of *Psa*-GFPuv in detached kiwifruit leaves display significant differences at different environmental conditions

**Figure 5:**
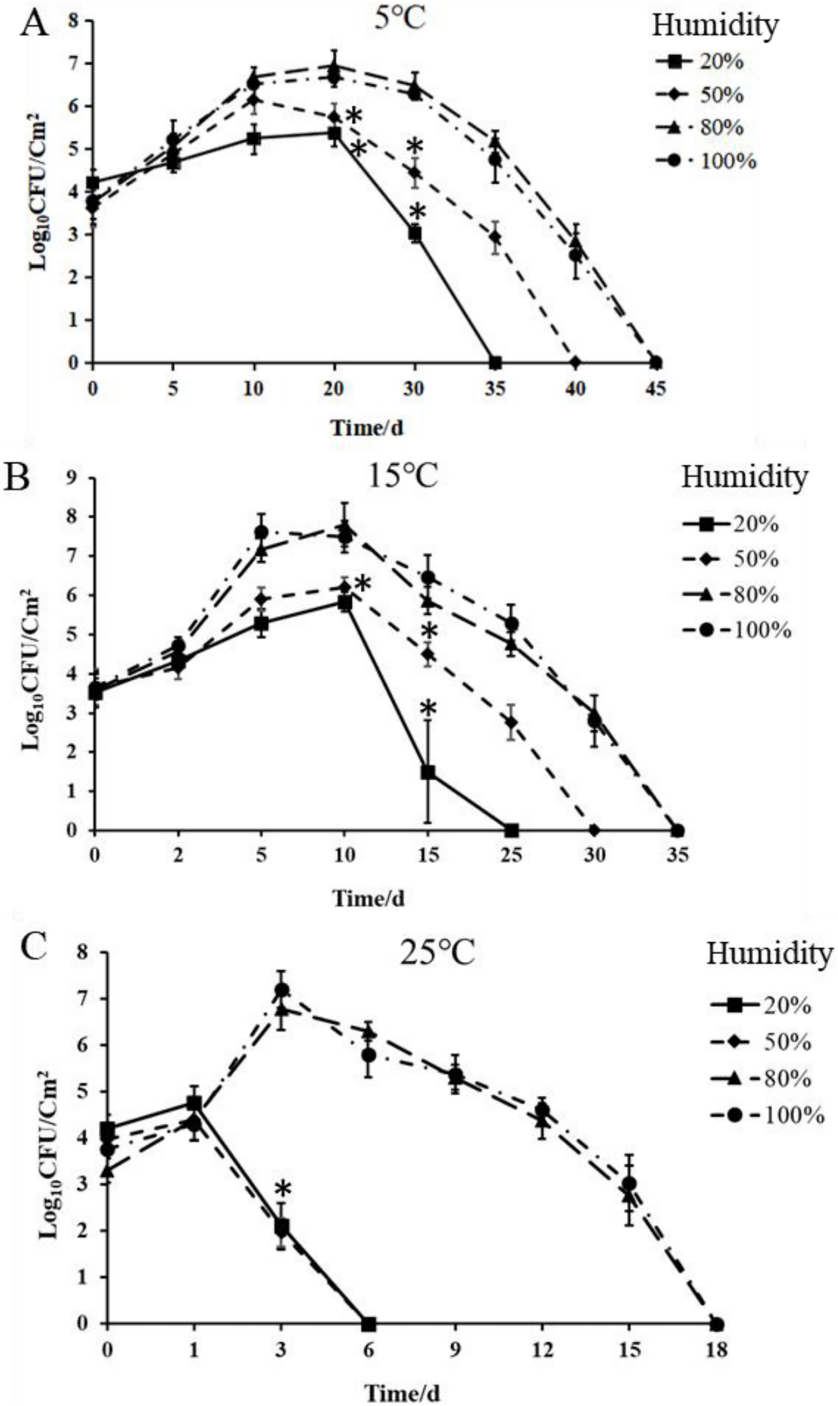
Effect of different temperatures and humidity on *Psa*-GFPuv bacterial growth in kiwifruit leaves. (A) Bacterial suspensions of *Psa*-GFPuv strain were injected into leaves of kiwifruit at 5 °C with different humidity. (B) At 15 °C with different humidity. (C) At 25 °C with different humidity. Experiments were repeated three times. Data represent the means of three replicates ± SDs (error bars). Statistically significant differences were determined by one-way ANOVA and *P*-values were <0.05 (an asterisk (*) indicates *P* < 0.05, the populations of *Psa*-GFPuv with 80% and 100% humidity *vs* the 20% and 50% humidity).

To quantitatively analyze the survival of bacteria in kiwifruit leaves under different environmental conditions conditions, *Psa*-GFPuv was inoculated into the detached kiwifruit leaves. Bacterial growth of the *Psa*-GFPuv strain was increased within 20 dpi and 10 dpi at 5 °C and 15 °C, respectively, and decreased within 20–45 dpi and 10–35 dpi (Figs. 5A and 5B). Moreover, bacterial growth of the *Psa*-GFPuv strain was decreased within 1–6 dpi with 20% and 50% humidity at 25 °C, whereas *Psa*-GFPuv strain populations were decreased within 3–18 dpi with 80% and 100% humidity (Fig. 5C). Furthermore, the populations of *Psa*-GFPuv strain with 80% and 100% humidity was significantly (*P* < 0.05) higher than 20% and 50% humidity at 5 °C, 15 °C and 25 °C, respectively (Figs. 5A–5C). These results further indicated that low temperature and high humidity conditions were more conducive to the survival and infection of *Psa* in kiwifruit leaves.

## Discussion

Since the kiwifruit canker was reported, researchers have mainly carried out work on the occurrence and control measures, and there is a lack of research on the movement path of *Psa* in kiwifruit. The infection path of *Psa* causing kiwifruit canker is still unclear. This leads to high costs and poor efficiency in the prevention and control of kiwifruit canker. In this study, we observed that the infection process of *Psa* in branches and leaves was longitudinal rather than transverse. Previous research found that the colonization sites of *Psa* in tissues are intracellular and intercellular spaces in the parenchyma of the cortex and phloem tissues (Renzi et al., 2015). In addition, *Psa* mainly migrates through the intercellular space (Huang, 2013). There are several layers of vascular formation between the phloem and the xylem that are tightly arranged with little space for cells. Therefore, *Psa* may be more susceptible to linear infection along the phloem than *Psa* from phloem to xylem. The longitudinal movement of *Psa* may explain why one side of the kiwifruit tree is symptomatic, while the other parts are healthy.

Previous reports found that the *Psa* can invade through stomata, hydathode and mechanical wounds, and then conduct to the petiole and stem or spread in the tissue by infecting branches, thus causing leaf disease (Serizawa, Ichikawa & Suzuki, 1994; Everett, 2011; Gao et al., 2016). Plant branches were inoculated with *Psa* in winter, and it was detected that *Psa* had migrated to a range of 2 cm above and below the inoculation site after 3 min (Ferrante & Scortichini, 2014). Root irrigation treatment of potted kiwifruit showed that marker bacteria could adsorb on the root surface, and invade the root system and proliferate, then after 2 weeks of root irrigation treatment, marker colonies were isolated from the stem tissue 4–6 cm away from the root (Huang, 2013). In this study, we mainly intended to simulate the survival state of *Psa* in fallen leaves under different environmental conditions, thus indicating that the incubation period of *Psa* is relatively long, and infected fallen leaves may be the source of infection in the next year. Therefore, the leaves of kiwifruit infected with *Psa* falling on soil may be an important source of infection to kiwifruit roots. We will focus further on this research in the future.

*Psa* mainly overwinter on diseased tissues, soil surfaces, and wild kiwifruit, then causing a new round of infection in the next year at a temperature suitable (Wang, Xiao & Wang, 2002). Previous studies have shown that *Psa* is mainly transmitted by wind and rain (Froud et al., 2015; Wang et al., 2022), and the average temperature of 10~20 °C is conducive to the outbreak of kiwifruit canker, while the temperature exceeding 25 °C will stop transmission (Serizawa & Ichikawa, 1993). A large number of field survey results show that the initial stage of kiwifruit canker occurrence is generally in the dormant stage of the plant, and the *Psa* begin to invade the plant by the stomata, lenticel and wounds of the plant (Serizawa et al., 1989; Zhong et al., 2020; Wang, Xiao & Wang, 2002). *Psa* can survive in moist soil for a long time, while extremely short in dry or plant-free soils (Everett, Pushparajah & Vergara, 2012). In addition, previously reported that different temperature was signficantly affected the colonization and movement of *Psa*-GFPuv in kiwifruit tissues (Gao et al., 2016). However, environmental humidity is also the main factor affecting the colonization and survival of *Psa* in kiwifruit tissues, so the influence of the superposition effect of temperature and humidity on the survival of *Psa* in kiwifruit tissues is still unknown. Therefore, in order to further clarify the infection and survival status of *Psa* under different temperature and humidity conditions. The survival time and colonization ability of bacteria in fallen leaves under different environmental conditions were simulated by the way of *Psa* infecting the detached kiwifruit leaves. In this study, our results showed that the survival time of *Psa* in detached kiwifruit leaves was the longest with above 80% humidity at 5 °C, and survival shortest with 20% humidity at 25 °C. Kiwifruit leaves quickly lost water with 20% humidity at 25 °C, which may be the reason for the short survival time of *Psa* in detached kiwifruit leaves. Therefore, these results indicated that *Psa* could survive in detached kiwifruit leaves for a long time under low temperature and high humidity conditions.

## Conclusions

*Psa* is hindered by plant tissue and spread mainly linearly rather than as a whole in kiwifruit plants. *Psa* survives longer in plant tissue under low temperature and high humidity. Among, the survival time of *Psa* in detached kiwifruit leaves was the longest with above 80% humidity at 5 °C. This research suggest that the survival condition of *Psa* in detached kiwifruit leaves is significantly affected by environmental conditions. Therefore, *Psa* has a long incubation period and tends to break out in fallen kiwifruit leaves when environmental conditions are suitable. The research on the infection path and survival time under different environmental conditions provides the basis for the control timing and the prevention strategy of kiwifruit canker.

## Supplemental Information

10.7717/peerj.15031/supp-1 Supplemental Information 1 The diagram of kiwi leaves grouped and sampled for quantitative analysis the bacterial growth.

10.7717/peerj.15031/supp-2 Supplemental Information 2 The raw data of survival time and populations of Psa inoculate kiwi leaves at different environmental conditions. The Fig. 4 data indicates the survival time of pathogens in detached kiwifruit leaves with different temperature and humidity. The Fig. 5 data indicates the quantitative analyze the survival of bacteria in kiwifruit leaves under different environmental conditions.

## Additional Information and Declarations

### Data Availability

The following information was supplied regarding data availability: The raw measurements are available in the Supplemental Files.
