# Supplementary figures and images for: Survival of Pseudomonas syringae pv. actinidiae in detached kiwifruit leaves at different environmental conditions
Source: PeerJ. 2023 Mar 10;11:e15031. doi: 10.7717/peerj.15031 (PMC10010172; doi:10.7717/peerj.15031)

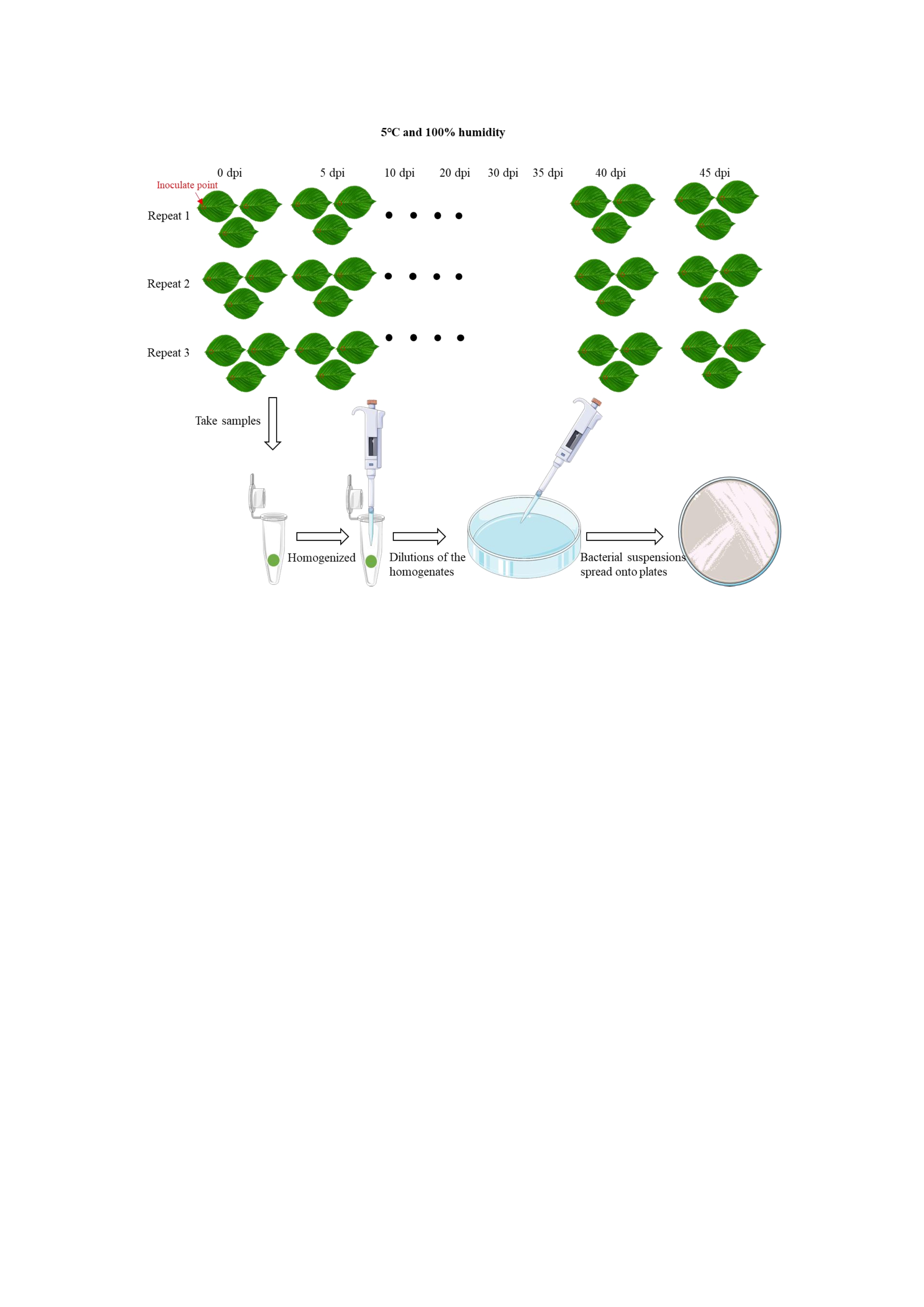

Supplement: Supplemental Information 1 [file peerj-11-15031-s001.jpg]
